# On the Effect of Chloroform on Muscular Fibre

**Published:** 1852-04

**Authors:** W. F. Barlow


*On the Effect of Chloroform on Muscular Fibre.* By W. F. Barlow, Esq., M. R. C. S.—Mr. Barlow has performed a series of experiments, which seem to show that chloroform rapidly exhausts muscular irritability, and in common with other agents inducing the same effects, conduces to the rapid stiffening called rigor mortis. He observes: "It has been shown by Nysten, in his ingenious researches, that a long time intervenes, as a general rule, between dissolution and this peculiar rigidity, during which time the muscles remain irritable; and that the rigor does not approach until the muscular irritability is either completely or nearly extinguished. This is, of course, a main fact. All kinds of muscular action prove the irritability of muscles, except rigor mortis; this, on the contrary, is a form of contraction which prevails not until muscular irritability is lost. After *excessive voluntary* action, such as that of the hunted animal, which though exhausted to the utmost, still runs for life, and forces to contraction muscles become almost too languid to respond effectually to any stimulus however violent, rigor mortis has been commonly noted to ensue with unusual rapidity. As Nysten says, "c'est parceque l'action vitale du liévre que le chasseur a forcé est, pour ainsi dire, epuisée par une fatigue excessive, que cette animal se roidit en mourant." So, too, *extreme voluntary* muscular contraction has been known to lead to the swiftest possible death-rigor. And it is very probable that the exhaustion of muscular irritability after death by galvinism would be found to hasten the phenomenon.

The experiments were performed on frogs and birds, decapitated and skinned, and afterwards suspended in a bottle containing chloroform. In a medico-legal point of view they are important, as if the same effects are produced on the human subject, a person poisoned with chloroform might, from the muscular rigidity be thought to have been dead some time, when in reality the reverse is the case.—*Medical Gazette*, Oct. 24, 1851.

